# The impact of physical exercise on esports players: a monitoring perspective

**DOI:** 10.3389/fpubh.2025.1558247

**Published:** 2025-02-17

**Authors:** Muhammet Kusan, Burhan Başoğlu, Mert Aydoğmuş, Sermin Ağralı Ermiş, Gülşah Sekban, Mustafa Tolga Bayraktar, Mehmet Vakıf Durmuşoğlu, Erkal Arslanoğlu, Faik Öz, Levent Ceylan, Hamza Küçük, Fatma Neşe Şahin

**Affiliations:** ^1^Yasar Doğu Faculty of Sport Sciences, Ondokuz Mayıs University, Samsun, Türkiye; ^2^Faculty of Sport Sciences, Nevsehir Haci Bektas Veli University, Nevşehir, Türkiye; ^3^Department of Coaching Education, Hasan Dogan Faculty of Sport Sciences, Karabuk University, Karabuk, Türkiye; ^4^Faculty of Sport Sciences, Adnan Menderes University, Aydın, Türkiye; ^5^Department of Coaching Education, Faculty of Sport Sciences, Sinop University, Sinop, Türkiye; ^6^Ministry of Ministry of National Education, Ankara, Türkiye; ^7^Institute of Graduate Studies, Ondokuz Mayıs University, Samsun, Türkiye; ^8^Faculty of Sport Sciences, Hitit University, Çorum, Türkiye; ^9^Department of Coaching Education, Faculty of Sport Sciences, Ankara University, Ankara, Türkiye

**Keywords:** esports players, physical exercise, physical health, body appreciation, esports

## Abstract

**Background:**

Esports has been defined as an organized and competitive approach to playing computer games. The esports industry has grown significantly and continues to develop in recent years. Within this period, monitoring and promoting physical exercise participation among esports players is important. The main aim of this study is to examine esports players’ participation in physical exercise. Another aim is to investigate the impact of physical exercise on body appreciation among esports players.

**Method:**

The study involved 183 esports players (age mean: 23.26; SD: 4.30; *n* = 42 female, *n* = 141 male). Data were collected using the Personal Information Form, the International Physical Activity Questionnaire Short Form (IPAQ), and the Body Appreciation Scale.

**Results:**

The analysis revealed significant differences in total physical activity and body appreciation scores and their sub-dimensions based on income level and years of esports licensing. Demographic factors such as gender, income level, and duration of esports participation were found to have notable effects on physical activity and body appreciation.

**Discussion:**

Male players had higher physical activity levels and body appreciation scores than females, and individuals with higher incomes reported better body appreciation. However, an increase in the duration of esports participation led to a decrease in physical activity levels and body appreciation scores. Increasing esports players’ participation in physical activity and promoting physical exercise are recommended.

## 1 Introduction

Digital games are considered a form of sports practice that involves individuals using their cognitive and physical abilities, either alone or in teams, within an electronic environment (1). Digital games are games that require attention, strategy, and coordination. These necessary conditions allow for the development of various skills in players (2). These effects can emerge in different age groups and populations. These effects may exhibit different developmental characteristics with the diversity of games and their changes over time. These developments can promote various perceptions and modifications over time.

Esports is a phenomenon that differs from traditional games and has gained popularity in recent years. Leagues have been established for esports, and tournaments are organized within these leagues (3). From a sports performance perspective, esports is considered both an emerging process and a tool for optimizing training and performance (4). In esports activities, participants do not engage in physical interventions. However, the excitement, motivation, and competition they experience are similar to the effects of physical activity (5).

It is important to monitor esports players’ participation in physical activity over specific periods and the challenges they encounter. Therefore, some studies have been conducted on this topic. Long et al. (6) developed a protocol to assess physical activity performance in esports players. Esnard et al. (7) stated that esports increased motivation in older adults through the acquisition of new gaming skills. Lemay et al. (8) highlights that esports players report positive consequences such as enhanced excitement, motivation, and competitive engagement, which parallel some benefits typically associated with physical activity, particularly in fostering mental stimulation and social connections. Therefore, monitoring esports players’ physical activity and promoting engagement is important. Scoggins et al. (9) stated that adults play video games 14 h more per week compared to 2018 (10). The increase in video game playtime suggests the need for interventions to encourage participation in physical activity.

However, the sedentary nature of esports participation may pose risks to physical health, potentially leading to a decrease in overall physical activity levels. A growing body of research suggests that prolonged gaming sessions could contribute to health problems typically associated with sedentary lifestyles, such as cardiovascular issues, obesity, and mental health challenges (11). Despite this, there is limited research specifically examining the relationship between esports participation and physical activity levels. The current study seeks to fill this gap by investigating the physical activity participation levels of esports players and exploring how physical activity correlates with body appreciation.

Therefore, the aim of this study is not only to measure esports players’ participation in physical activity but also to examine the relationship between this participation and body appreciation. This research contributes to the growing understanding of the health implications of esports participation and provides valuable insights for promoting better health practices among esports players.

## 2 Materials and methods

### 2.1 Research group

A total of 183 esports players voluntarily participated in the study. Of the participants, 23% were female (*n* = 42) and 77% were male (*n* = 141). In the study, approval was obtained from the Ethics Committee of Social and Human Sciences at Ondokuz Mayıs University, with the decision numbered 2024–808, to administer the scales and collect the data.

### 2.2 Data collection tools

As data collection tools, the Personal Information Form, the International Physical Activity Questionnaire Short Form (IPAQ-SF), and the Body Appreciation Scale were utilized.

The International Physical Activity Questionnaire (IPAQ-SF) was developed by Craig et al. (12) with the support of the World Health Organization (WHO) and the Centers for Disease Control and Prevention (CDC). Its validity and reliability study for Turkey was conducted by Saglam et al. (13). This form measures the time individuals spend on light, moderate, and vigorous physical activities as well as their sedentary time. For evaluation, each activity must be performed for at least 10 min continuously. MET (Metabolic Equivalent) values for each activity level are multiplied by the duration (in minutes) and frequency (in days) to calculate a “MET-min/week” score. These scores are categorized based on physical activity levels as inactive, low active (MET <600), moderately active (MET = 600–3,000), and highly active (MET >3,000). To determine the energy expenditure for each activity, the weekly duration (in minutes) of that activity is multiplied by its MET value specific to IPAQ.

The Body Appreciation Scale was developed by Avalos et al. (14) to measure individuals’ levels of body appreciation. Its Turkish adaptation was conducted by Bakalım and Taşdelen-karçkay (15), resulting in the Turkish version of the scale. The scale consists of 9 items and follows a 5-point Likert-type structure. The minimum score is 9, and the maximum score is 45. Confirmatory factor analysis revealed that the scale has a two-factor structure. Analyses demonstrated that the scale has sufficient validity and internal consistency, as well as composite reliability. The Cronbach’s alpha coefficient for internal consistency was calculated as 0.92. Higher scores indicate higher levels of body appreciation. The scale does not include any reverse-scored items. Items 1, 2, 3, 4, 5, 8, and 9 reflect the first factor, general body appreciation, while items 6 and 7 represent the second factor, investment in body image (15).

### 2.3 Data collection

Before administering the survey questions to the e-athletes constituting the research group, the purpose of the study was explained.

### 2.4 Statistical analysis

In the study, reliability coefficients (Cronbach’s alpha) were calculated to assess the internal consistency of the responses given by individuals to the scale items (Table 1).

**Table 1:** Internal consistency coefficients for participants’ responses to scale ıtems.

Scale	Internal consistency coefficient	Evaluation
Body appreciation scale	0.938	Highly reliable
General body appreciation	0.922	Highly reliable
Body image investment	0.777	Moderately reliable

In the statistical evaluation of the data, the normality assumption was initially interpreted through the Kolmogorov–Smirnov test, the Shapiro–Wilk normality test, and skewness-kurtosis coefficients. In this study, whether the total scale scores differed by gender was determined using an independent samples t-test, while differences based on income level and E-sports license age were analyzed using One-Way Analysis of Variance (ANOVA) and Tukey’s *post hoc* test. Participants’ physical activity levels based on gender, income, and E-sports license age were determined using the Chi-square test. Furthermore, the direction of the relationship between participants’ body mass index, physical activity level, and body appreciation level was calculated using the Pearson correlation coefficient, and the equation was determined through regression analysis. All statistical calculations were performed using the SPSS 26.0 statistical software package. Research findings are presented as *n*(%), mean, and standard deviation values, and results were considered statistically significant at the *p* < 0.05 level.

## 3 Results

**Figure 1:**
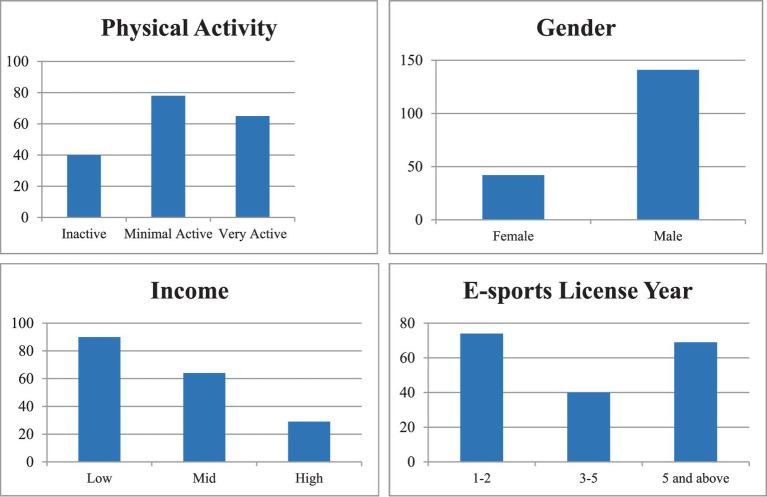
Frequency and percentage distributions of participants’ demographic characteristics.

Among the individuals who voluntarily participated in the study, 49.2% had a low income, 40.4% had an E-sports license age of 1–2 years, 23.0% were female, and 21.9% were inactive individuals (Figure 1).

The physical activity levels of participants vary by gender, income, and license year. While 7.1% of females are very active, 44% of males fall into the very active category. Among those with low income, 37.8% are inactive, whereas more than half of the players with medium and high income are very active. The very active rate is highest (52.7%) among participants with 1–2 years of license ownership. As the license duration increases (3–5 years and over 5 years), the proportion of very active participants decreases, while increases are observed in the inactive and minimally active categories (Table 2).

**Table 2:** Physical activity levels of participants by gender, income, and e-sports license year.

	Short international physical activity questionnaire					
	Inactive	Minimal active	Very active	Total		
Gender	*n* (%)	*n* (%)	*n* (%)	*n* (%)	χ^2−^	*p*
Female	17 (40.5)	22 (52.4)	3 (7.1)	42 (100)	22.220	<0.001
Male	23 (16.3)	56 (39.7)	62 (44)	141 (100)		
Income	*n* (%)	*n* (%)	*n* (%)	*n* (%)		
Low	34 (37.8)	47 (52.2)	9 (10)	90 (100)	60.183	<0.001
Mid	4 (6.3)	25 (39.1)	35 (54.7)	64 (100)		
High	2 (6.9)	6 (20.7)	21 (72.4)	29 (100)		
E-sport license age	*n* (%)	*n* (%)	*n* (%)	*n* (%)		
1–2	8 (10.8)	27 (36.5)	39 (52.7)	74 (100)	18.791	<0.001
3–5	12 (30)	20 (50)	8 (20)	40 (100)		
5 and above	20 (29)	31 (44.9)	18 (26.1)	69 (100)		

*n* (%) = Frequency (percentage); χ^2^ = Chi-square test statistic.

In the study, a statistically significant difference was found between the total scale scores and sub-dimension total scores of physical activity and body appreciation based on gender among esports players (*p* < 0.05; Table 3). Male players scored higher in physical activity and body appreciation compared to female players (Table 3).

**Table 3:** Physical activity and body appreciation levels of esports players by gender.

Scales and sub-dimensions	Gender	*n*	Mean	SD	*p*
International physical activity questionnaire	Female	42	713.01	531.08	<0.001
	Male	141	1609.18	1097.51	
Body appreciation scale	Female	42	28.10	10.35	0.015
	Male	141	32.06	8.80	
General body appreciation	Female	42	21.48	8.46	0.015
	Male	141	24.70	7.11	
Body image investment	Female	42	6.62	2.11	0.033
	Male	141	7.37	1.94	

A statistically significant difference was found between the total scale scores and sub-dimension total scores of physical activity and body appreciation among esports players based on income level (*p* < 0.05; Table 4). It was observed that as income level increased, physical activity levels also increased, and players with higher income levels had higher body appreciation scores compared to those with lower income levels (Table 4).

**Table 4:** Physical activity and body appreciation levels of esports players by income.

Scales and sub-dimensions	Income	*n*	Mean	SD	*p*
International physical activity questionnaire	Low	90	829.29c	582.87	<0.001
	Mid	64	1734.44b	988.77	
	High	29	2455.21a	1276.10	
Body appreciation scale	Low	90	27.37b	9.37	<0.001
	Mid	64	34.14ab	7.90	
	High	29	36.31a	7.03	
General body appreciation	Low	90	20.80b	7.55	0.003
	Mid	64	26.44ab	6.38	
	High	29	28.28a	5.58	
Body image investment	Low	90	6.57b	2.03	0.012
	Mid	64	7.70ab	1.80	
	High	29	8.03a	1.76	

A statistically significant difference was found between the total scale scores and sub-dimension total scores of physical activity and body appreciation among esports players based on license year (*p* < 0.05; Table 5). It was observed that as the license year increased, physical activity and body appreciation scores decreased (Table 5).

**Table 5:** Physical activity and body appreciation levels of esports players by income license year.

Scales and sub-dimensions	License year	*n*	Mean	SD	*p*
International physical activity questionnaire	1–2	74	1828.84a	1143.30	<0.001
	3–5	40	987.00b	611.93	
	5 and above	69	1188.79b	1033.65	
Body appreciation scale	1–2	74	33.80a	7.96	0.004
	3–5	40	28.63b	8.53	
	5 and above	69	29.78b	10.42	
General body appreciation	1–2	74	26.16a	6.34	0.005
	3–5	40	21.85b	6.94	
	5 and above	69	22.81b	8.49	
Body image investment	1–2	74	7.64a	1.90	0.044
	3–5	40	6.78b	1.85	
	5 and above	69	6.97b	2.13	

In the study, a moderate negative correlation was found between individuals’ BMI and physical activity scores (r = −0.414). A low negative correlation was observed between BMI and the total body appreciation scale score (r = −0.350), general body appreciation (r = −0.346), and the sub-dimension score of Investment in Body Image Investment (r = −0.324). Additionally, a moderate positive correlation was found between total physical activity scores and the total body appreciation scale score, as well as sub-dimension scores (Table 6).

**Table 6:** Correlation table for the relationship between participants’ body mass index, physical activity level, and body appreciation level.

		PAS	BAS	GBA	BII
BMI	*r*	−0.414	−0.350	−0.346	−0.324
	*p*	<0.001	<0.001	<0.001	<0.001
FAS	*r*		0.544	0.553	0.443
	*p*		<0.001	<0.001	<0.001

BMI = Body Mass Index; PAS = Physical Activity Total Score; BAS = Body Appreciation Total Score; GBA = General Body Appreciation; BII = Body Image Investment.

The regression analysis revealed a significant regression model between physical activity and body appreciation scale. The change in physical activity level explains 29.5% (R^2^ = 0.295) of the variation in body appreciation. 1-unit increase in esports players’ physical activity score will lead to a 0.005-point increase in their body appreciation score (Table 7).

**Table 7:** Regression analysis results on the effect of participants’ physical activity level on body appreciation.

Variables	*B*	SE	*R^2^*	*t*	*P*
Constant	24.485	0.960	0.295	25.512	<0.001
Physical activity	0.005	0.001		8.711	<0.001

B = Unstandardized regression coefficient; SE = Standard Error; R^2^ = Coefficient of determination; t = t-statistic.

## 4 Discussion

This study was conducted to examine the levels of physical activity and body appreciation among active e-sports players. The research identified significant differences in total physical activity and body appreciation scores, as well as subdimension scores, based on income level and years of e-sports licensing.

The results of the study indicate that the physical activity levels of e-sports players show significant differences depending on gender, income, and licensing duration. While 44% of male participants were classified as very active, only 7.1% of female participants were at this level. This discrepancy may be associated with societal norms and structural barriers that hinder women’s participation in physical activities (16, 17). Income level also appears to have a noticeable impact on physical activity. Among low-income players, 37.8% were classified as inactive, whereas more than half of the players in middle and high-income groups fell into the very active category. These results highlight the role of financial resources in accessing physical activity and underscore the importance of economic support mechanisms for low-income individuals. For example, free access to sports facilities or community-based sports events can help reduce these inequalities (17). Licensing duration is another significant determinant of differences in physical activity levels. According to the study, 52.7% of players with 1–2 years of licensing were very active, but this percentage decreased as the licensing duration increased, with more players becoming inactive or minimally active over time (18). The intensity of participation in esports may vary, with younger players typically being more engaged. Over time, novelty may wear off, or players may face increased life responsibilities, which could lead to a decrease in activity (19, 20). This trend suggests that initial enthusiasm may wane over time or that sedentary behaviors may replace physical activity as licensing duration increases (11). Factors such as academic pressures or spending more time on e-sports may contribute to this decline. However, despite these findings, it is noted that different areas of physical activity need to be evaluated. Some studies suggest that differences based on variables such as gender and income may not be as pronounced when all types of physical activities are considered (21). Therefore, targeted interventions designed to increase the physical activity levels of e-sports players, considering demographic differences like gender and income and aligned with licensing durations, are necessary. Holistic approaches to physical activity can support both the physical and psychological well-being of players.

The study found statistically significant differences in the physical activity levels and body appreciation scale scores of e-sports players based on gender. Male players scored higher in both physical activity levels and body appreciation. This finding aligns with broader literature examining the impact of gender differences on physical activity and body appreciation (22, 23). The tendency of male players to report higher physical activity levels may result from a combination of factors. Males may participate more in physical activities outside of gaming, reflecting both self-report biases and actual differences in activity levels (23). The higher body appreciation scores among male players may be a result of societal pressures and differing body image standards between genders. Men are generally more inclined to have positive perceptions regarding muscle structure and physical appearance, whereas women may be more affected by societal pressures concerning body image (22).

As income level increases, physical activity levels rise, and players with higher incomes report higher body appreciation scores compared to those with lower incomes. The relationship between income level and physical activity reveals important trends affecting body appreciation among e-sports players. Higher-income players tend to report better body appreciation, which may be associated with greater access to fitness programs and nutrition resources (23). These financial opportunities may increase intrinsic motivation for physical activity and indirectly create positive effects on body image (24–26). However, it should not be overlooked that sedentary behaviors are prevalent among e-sports players, regardless of income level, and that all players may not prioritize physical activity (27).

The study identified a statistically significant difference in total and subdimension scores for physical activity and body appreciation scales based on licensing year. As licensing years increased, physical activity and body appreciation scores decreased. This trend highlights that longer participation in e-sports is associated with decreased physical engagement and body appreciation, emphasizing the potential health implications for players. E-sports players generally report lower levels of physical activity, with one study indicating that players engage in an average of only 18.2 min of moderate-to-vigorous physical activity per day (25). In contrast, it was noted that players tend to spend an average of 573.4 min per day sitting, demonstrating a sedentary lifestyle (25). The decline in body appreciation may be linked to increased sedentary behaviors and a higher body mass index (BMI), with e-sports players having an average BMI of 26.03 (28). Players with longer e-sports experience may develop negative body image perceptions due to prolonged inactivity (23). However, some research suggests that e-sports participants engage more with traditional sports and non-gaming activities, indicating that not all e-sports participation leads to negative health outcomes (29).

The relationships between BMI, physical activity, and body appreciation provide valuable insights into health behaviors among individuals. The study found a moderate negative relationship between BMI and physical activity scores (r = −0.414). This suggests that as BMI increases, physical activity levels tend to decrease. This finding aligns with existing literature, which associates higher BMI with limitations in physical mobility, social stigma, or decreased motivation to participate in physical activity (30). Additionally, low-level negative correlations were found between BMI and overall body appreciation (r = −0.346) and Body Image Investment (r = −0.324). This indicates that individuals with higher BMI are more likely to experience disappreciation with their physical appearance. Societal beauty standards and internalized body perceptions may exacerbate this disappreciation. These findings emphasize the need for interventions that promote positive body image and mental well-being, regardless of BMI. Moreover, the positive moderate relationship detected between physical activity scores and overall body appreciation (r = 0.350) suggests that increased physical activity can enhance body appreciation perceptions. This underscores the positive impact of active lifestyles on both physical and psychological health (31, 32).

Regression analysis revealed a significant relationship between physical activity and body appreciation, explaining 29.5% of the variance in body appreciation scores. It was found that each unit increase in physical activity scores led to a 0.005-point increase in body appreciation, indicating that individuals’ body appreciation improves as they increase their physical activities. This finding is consistent with previous studies highlighting the importance of physical activity in improving body perceptions and health outcomes (33). Physical activity during adolescence, in particular, plays a critical role in shaping body appreciation and developing positive body image perceptions. It not only enhances body appreciation but also contributes to overall mental health, reducing anxiety and depression (34). Continuous physical activity can lead to long-term improvements in body image, especially for individuals experiencing self-esteem issues during adolescence (35). However, it should be noted that the relationship between physical activity and body appreciation is not equally strong across all demographic groups. Variables such as gender, socioeconomic status, or cultural factors can influence this relationship (34). These findings clearly demonstrate that while promoting physical activity is essential, interventions should be tailored to individuals’ demographic characteristics and needs.

In conclusion, this study provides significant insights into the physical activity levels, body appreciation, and relationships between these variables among e-sports players. Demographic factors such as gender, income level, and e-sports participation duration have distinct impacts on physical activity and body appreciation. Male players reported higher physical activity levels and body appreciation scores compared to females, while higher-income individuals reported better body appreciation. However, increased e-sports participation duration was associated with declines in both physical activity levels and body appreciation scores. Regression analysis highlighted the significant effect of physical activity on body appreciation, emphasizing the importance of promoting physical activity to improve body perceptions. Additionally, the identified relationships between BMI, physical activity, and body appreciation underscore the necessity of encouraging healthy lifestyle habits. These findings suggest that interventions promoting physical activity should be adapted to individuals’ demographic characteristics to support their overall physical and psychological well-being.

### 4.1 Recommendations

To support the health and well-being of e-sports players, promoting physical activity and reducing the effects of a sedentary lifestyle are of critical importance. Programs incorporating regular breaks for physical activity can be developed, and awareness campaigns aimed at improving body appreciation, particularly for female players, through gender-specific approaches can be organized. Community-based initiatives and sponsorships should be provided to facilitate access to sports facilities for low-income players. Additionally, guidance and education should be offered to balance the negative health effects of long-term e-sports participation. Physical activity-promoting events and rewards can be integrated into e-sports tournaments. These interventions, designed with cultural and individual differences in mind, can be combined with counseling services that support mental health and body image, offering a comprehensive approach to enhancing both the physical and psychological well-being of players.

## Data Availability

The original contributions presented in the study are included in the article/supplementary material, further inquiries can be directed to the corresponding author.
